# Calcium signals in the nucleus accumbens: Activation of astrocytes by ATP and succinate

**DOI:** 10.1186/1471-2202-12-96

**Published:** 2011-10-03

**Authors:** Tünde Molnár, Árpád Dobolyi, Gabriella Nyitrai, Péter Barabás, László Héja, Zsuzsa Emri, Miklós Palkovits, Julianna Kardos

**Affiliations:** 1 Department of Neurochemistry, Institute of Biomolecular Chemistry, Chemical Research Center, Hungarian Academy of Sciences, Pusztaszeriút 59-67, 1025 Budapest, Hungary; 2 Neuromorphological and Neuroendocrine Research Laboratory, Semmelweis University and Hungarian Academy of Sciences, Tűzoltó utca 58, 1094 Budapest, Hungary; 3 Department of Ophthalmology, University of Utah, 65 Mario Capecchi Drive, Salt Lake City, UT 84132, USA; 4 Department of Zoology, Eszterházy Károly College, Leányka utca 6, 3300 Eger, Hungary

## Abstract

**Background:**

Accumulating evidence suggests that glial signalling is activated by different brain functions. However, knowledge regarding molecular mechanisms of activation or their relation to neuronal activity is limited. The purpose of the present study is to identify the characteristics of ATP-evoked glial signalling in the brain reward area, the nucleus accumbens (NAc), and thereby to explore the action of citric acid cycle intermediate succinate (SUC).

**Results:**

We described the burst-like propagation of Ca^2+ ^transients evoked by ATP in acute NAc slices from rat brain. Co-localization of the ATP-evoked Ca^2+ ^signalling with immunoreactivities of the astroglia-specific gap junction forming channel protein connexin43 (Cx43) and the glial fibrillary acidic protein (GFAP) indicated that the responsive cells were a subpopulation of Cx43 and GFAP immunoreactive astrocytes. The ATP-evoked Ca^2+ ^transients were present under the blockade of neuronal activity, but were inhibited by Ca^2+ ^store depletion and antagonism of the G protein coupled purinergic P2Y_1 _receptor subtype-specific antagonist MRS2179. Similarly, Ca^2+ ^transients evoked by the P2Y_1 _receptor subtype-specific agonist 2-(Methylthio)adenosine 5'-diphosphate were also blocked by MRS2179. These characteristics implied that intercellular Ca^2+ ^signalling originated from the release of Ca^2+ ^from internal stores, triggered by the activation of P2Y_1 _receptors. Inhibition by the gap junction blockers carbenoxolone and flufenamic acid and by an antibody raised against the gating-associated segment of Cx43 suggested that intercellular Ca^2+ ^signalling proceeded through gap junctions. We demonstrated for the first time that extracellular SUC also evoked Ca^2+ ^transients (EC_50 _= 50-60 μM) in about 15% of the ATP-responsive NAc astrocytes. By contrast to glial cells, electrophysiologically identified NAc neurons surrounded by ATP-responsive astrocytes were not activated simultaneously.

**Conclusions:**

We concluded, therefore, that ATP- and SUC-sensitive Ca^2+ ^transients appear to represent a signalling layer independent of NAc neurons. This previously unrecognised glial action of SUC, a major cellular energy metabolite, may play a role in linking metabolism to Ca^2+ ^signalling in astrocytic networks under physiological and pathological conditions such as exercise and metabolic diseases.

## Background

In astrocytes of the brain reward area, the nucleus accumbens (NAc; [1]), γ-hydroxybutyric acid (GHB; [2]) evoked intracellular store-reliant Ca^2+ ^transients, independently of neuronal activity [3]. Previously, we also showed that binding sites for GHB are shared with citric acid cycle intermediate succinic acid (SUC) and the gap-junction blocker carbenoxolone hemisuccinate (CBX), as disclosed in NAc membrane homogenates isolated from rat and human brain tissues [4-6]. These findings raised the possibility that SUC, similarly to GHB may also evoke Ca^2+ ^transients in NAc astrocytes. Further, it is conceivable that the rather specific sensitivity of the SUC/GHB target site to CBX might be a sign of its functional association with connexin channels. In order to study the effect and functional significance of SUC on the Ca^2+ ^homeostasis of NAc astrocytes, we considered that the Ca^2+ ^bursting activity was found ATP-responsive in vivo, *i.e*. in Bergmann glia networks activated by the motor behaviour of the awaken animal [7]. Therefore, we sought to characterise first the ATP-responsive Ca^2+ ^signalling amongst the astrocytes of the NAc.

ATP is known to evoke Ca^2+ ^bursts by activation of purinergic G-protein-coupled receptors (GPCRs) *in vitro *[8-10] as well as *in vivo *[7,11-13]. Different *in vitro *paradigms, including locally administered ATP stimuli (100 μM) were found effective to evoke Ca^2+ ^transients [14-18]. In the present study, we investigated if locally ejected ATP (100 μM) could evoke Ca^2+ ^bursting in NAc astrocytes. Measurements were performed by combined application of confocal Ca^2+ ^imaging, immunohistochemistry and electrophysiology in acute NAc tissue slices prepared from the rat brain. Astrocytes were identified by co-localization of astrocyte-specific antibodies raised against the astroglial gap-junction protein connexin 43 (Cx43) and the glial fibrillary acidic protein (GFAP). Then, ATP-evoked Ca^2+ ^bursts have been characterised by using of various drugs and agents, including gap-junction inhibitors (CBX, flufenamic acid: FFA), an antibody raised against the gating peptide segment of Cx43, purinergic P2 receptor agents such as the broad-spectrum P2X and P2Y receptor antagonist suramin (SUR), P2Y_1 _subtype-specific agonist 2-(Methylthio)adenosine 5'-diphosphate (2-Me-S-ADP) and antagonist MRS2179, the Na^+ ^channel blocker tetrodotoxin (TTX) and the Ca^2+ ^store depleting cyclopiazonic acid (CPA). Moreover, we also demonstrate for the first time the existence of SUC-responsive Ca^2+ ^transients that overlay in a sub-population of NAc astrocytes.

## Results

In selecting the NAc region of interest, we first considered area-dependent distribution of Cx43 protein and its co-localization with GFAP. Next, we asked if Cx43-positive NAc astrocytes were responded to local administration of ATP by Ca^2+ ^transients. Subsequently, the hypothesis that SUC may also activate Ca^2+ ^transients playing part in the ATP-responsive Ca^2+ ^signals was tested. Finally, responsiveness of NAc neurons to ATP was explored.

### Demonstration of ATP-evoked burst-like Ca^2+ ^signals amongst astrocytes in the NAc

**Figure 1:**
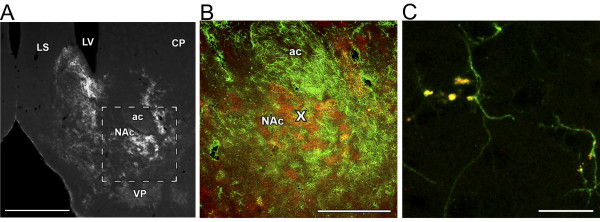
**Cx43 is present in GFAP-containing astrocytes in the NAc**. **A**: Cx43 immunoreactivity in a coronal section of a PFA-fixed rat brain shows relatively high intensity in the NAc, as compared to adjacent brain regions. Abbreviations: ac - anterior commissure, CP caudate-putamen, LS - lateral septal nucleus, LV - lateral ventricle, NAc - nucleus accumbens, VP - ventral pallidum. Scale bar: 1 mm. **B: **Comparison of the appearance of Cx43 (red) and GFAP (green) proteins in the NAc. Low magnification (scale bar: 400 μm) confocal photomicrograph of a double labelled section of a PFA-fixed rat brain. Co-localization (yellow) of Cx43 (red) and GFAP (green) immunoreactivities in the NAc suggests that Cx43 is present in GFAP-containing astrocytes in this brain region. Label "x" indicates the site of ATP application. C: High magnification (scale bar: 20 μm) confocal photomicrograph of a double labelled section of a PFA-fixed rat brain. Yellow colour indicates co-localizations of Cx43 (red) and GFAP (green) immunoreactivities in the PFA-fixed NAc.

The expression and distribution of Cx43 have been first characterised in rat brain sections by immunohistochemistry. The staining pattern in a coronal section of a paraformaldehyde (PFA)-fixed rat brain revealed intense staining in both the shell and the core regions in the NAc (Figure 1A). High intensity of Cx43 immunolabelling was observed in the NAc as compared to adjacent brain regions (including the caudate putamen), suggesting a relatively higher level of connectivity of the astrocyte network in the NAc through this type of gap-junctions. Besides the NAc, relatively high Cx43 immunolabelling was also found in other brain areas, including several viscerosensory and limbic regions: i) medial prefrontal cortex, ii) Purkinje cell layer of the cerebellar cortex, iii) hippocampus, particularly the molecular layer of the dentate gyrus, iv) central nucleus of the amygdala, v) dorsal subdivision of the lateral septal nucleus, vi) dorsolateral subdivision of the periaqueductal gray, and vii) nucleus of the solitary tract (data not shown). Cx43 had a patchy distribution in the NAc; that is, areas with high and low intensity of labelling were intermingled (Figure 1B). Where the labelling of Cx43 was intense, the vast majority of Cx43 cells contained astrocyte marker protein GFAP immunoreactivity as shown in Figure 1C.

**Figure 2:**
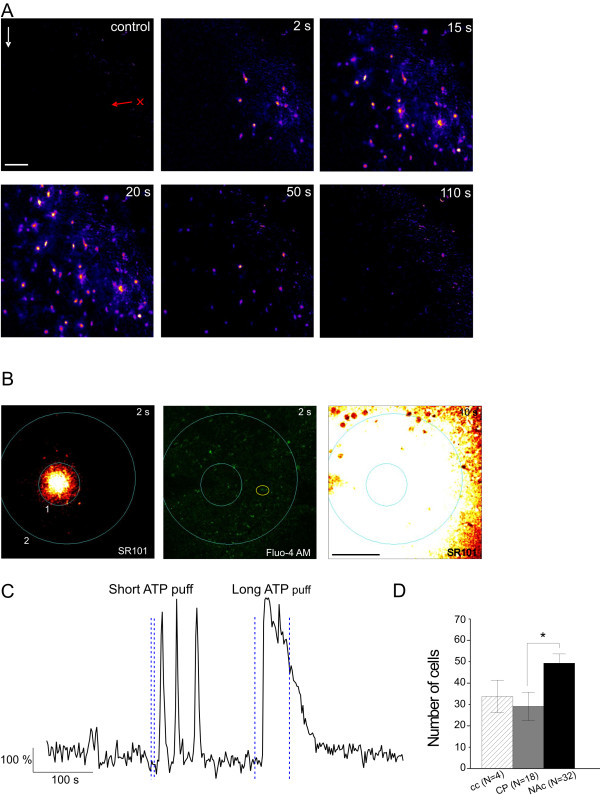
**Demonstration of ATP-responsive astroglial Ca^2+ ^signalling in the NAc slice**. A: Time-series of Fluo-4 pseudo-coloured fluorescence images (*see *also Additional file 1) show propagation of Ca^2+ ^transients induced by pressure-ejection of 100 μM ATP onto the surface of the NAc slice through a 5-10 μm patch-pipette for 60 s (long puff). Time refers to the period passed from the beginning of the long ATP puff. Arrows indicate the origin and orientation of the ATP puff (red with X) and the direction of ACSF perfusion (white). Scale bar: 50 μm. **B: **The area initially covered by the short (left and middle) and long (right) ATP puffs was investigated to observe which cells get a direct ATP stimulus. Representative images of the glial marker dye SR101 (100 μM dissolved in ACSF; [19,20]) applied together with 100 μM ATP to visualize propagation of ATP puff above 630 nm using a 543 nm laser for excitation. This was done in parallel with monitoring the ATP-responsive Fluo-4 Ca^2+ ^transients in the NAc slice. The small azure circle indicates the border of the ATP+SR101 puff for 2 s (short puff) and the big azure circle shows the margin of the ATP+SR101 diffusion. Yellow circle beyond the small azure circle highlight the cell responded to the short puff application of ATP. Scale bar: 100 μm. **C: **Representative (dF/F_0_)_max _Fluo-4 fluorescence plot of the cell highlighted by the yellow circled cell in panel **B **recorded during ATP application for 2 s (short puff) and for 60 s (long puff, *cf*. **B**). **D**: Number of networking cells in long ATP puff-stimulated Ca^2+ ^transients was significantly higher in the NAc when compared to the adjacent ventral pallidal area.

In freshly prepared NAc slices from 10-14 day old male rats, 100 μM ATP applied locally for 60 sec (long ATP-puff) evoked Ca^2+ ^transients, propagating in up to 127 cells (44 ± 25 in average) in a total of 212 slices from 53 rats (Figure 2A, additional file 1). In order to characterise the dynamics of Ca^2+ ^transients, short (2 s, Figure 2B left and middle) and long ATP (60 s, Figure 2B right) puffs were compared. The period of repetitive Ca^2+ ^transients observed after the application of the short ATP-puff (2 s) occurred on a time scale similar to that of the bulky Ca^2+ ^transient evoked by the long ATP-puff (Figure 2C). The observation suggested that the bulky Ca^2+ ^signal can be decomposed into many repetitive Ca^2+ ^transients providing kinetic evidence for a burst-like, coupled Ca^2+ ^dynamics. The number of ATP responsive astrocytes was significantly higher in the NAc compared to the adjacent ventral pallidal area (Figure 2D). In line with the Cx43 expression pattern (Figure 1), the observation conjectured Cx43-related ATP-responsive Ca^2+ ^signalling in the NAc. The burst-like Ca^2+ ^signal, apparently not influenced by the direction of the perfusion, propagated in round or ellipsoidal waveform at an approximate 10 μm/s speed (Figure 2A, Additional file 1). When monitored by the glial marker dye SR101 [19,20], the diffusion of ATP was about 1.5 times faster than the speed of the Ca^2+ ^wave propagation (Figure 2B left and middle). It is to note, that the dynamics of burst-like Ca^2+ ^signals of NAc astrocytes can be compared with that of Ca^2+ ^bursts of Bergmann glia in vivo [7,13].

### Molecular dissection of ATP-evoked burst-like Ca^2+ ^signalling

**Figure 3:**
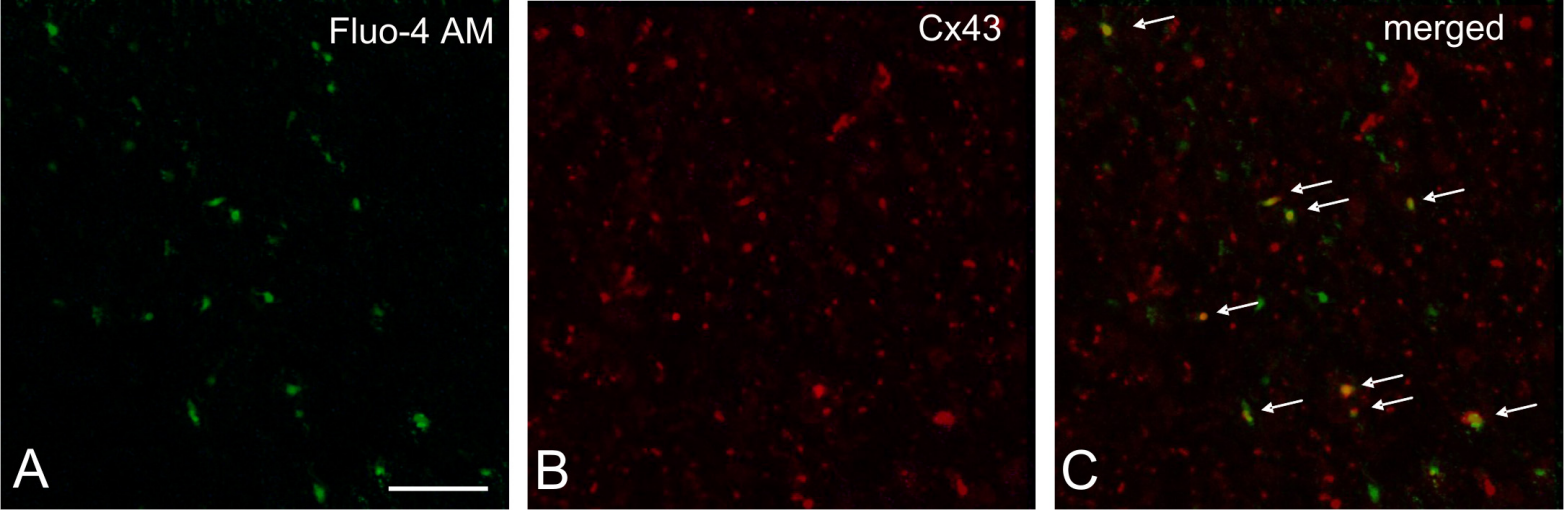
**Co-localization of ATP-evoked Ca^2+ ^transients with Cx43 immunostaining in the NAc slice**. Representative images of Ca^2+ ^signalling in response to the long ATP puff (green, left) followed by *post-calcium *(*cf*. Methods section) Cx43 immunostaining (red, middle) demonstrated their co-localization (yellow spots marked with white arrows, right). Scale bar: 50 μm.

In the lack of specific gap-junction blocker, we applied an antibody against the gating peptide segment of Cx43 [21] to inhibit Cx43 function and therefore to evaluate the involvement of gap-junctions in the ATP-evoked burst-like Ca^2+ ^signals. Few co-localization of Ca^2+ ^transients and Cx43 immunoreactivity was observed when NAc slices were *pre*-incubated with the Cx43 antibody (7.5 ± 2.3% of the ATP responsive cells; *N *= 6 from 2 rats), suggesting that inhibition of Cx43 function may preclude Cx43 immunoreactive astrocytes to participate in the Ca^2+ ^signal. In contrast, an about fourfold increase in co-localization of Ca^2+ ^transients and Cx43 immunoreactivity was seen (Figure 3; 31 ± 3% of the ATP responsive cells; *N *= 4 from 4 rats), when the same antibody was applied *after *the application of ATP (*cf*. Materials and Methods). This observation indicated that the blockade of the gap-junction could prevent ATP from evoking burst-like Ca^2+ ^signalling. Co-localizations of Ca^2+ ^transients with Cx43 (Figure 3) and those of Cx43 with GFAP (Figure 1B) conclusively suggested that the ATP-evoked burst-like Ca^2+ ^signalling occurred in NAc astrocytes.

**Figure 4:**
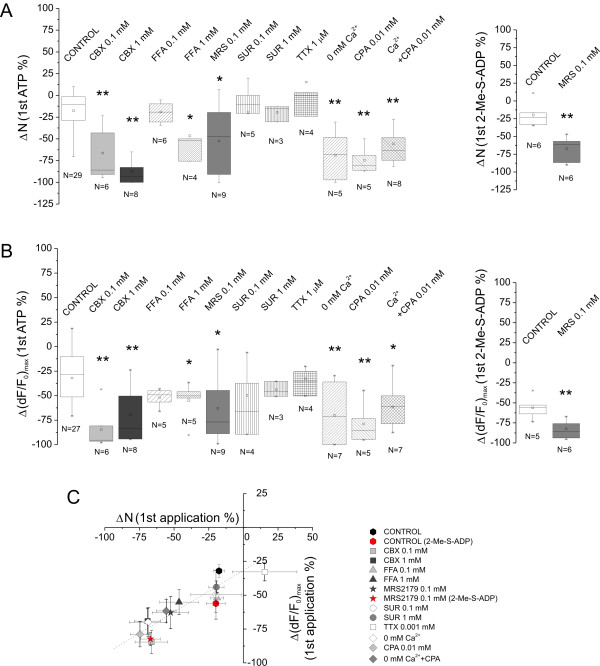
**Molecular dissection of ATP-evoked burst-like Ca^2+ ^signalling in the NAc slice**. A: Cell number-based profiling. Left - ATP-evoked of Ca^2+ ^transients: Effects of gap-junction blockers CBX, FFA; P2 antagonists SUR and MRS2179; Na^+ ^channel blocker TTX; Ca^2+ ^store depletor CPA with/without added external Ca^2+^. Right - 2-Me-S-ADP evoked Ca^2+ ^transients: Effect of MRS2179. B: Fluorescence intensity-based profiling. Left - ATP-evoked of Ca^2+ ^transients: Effects of gap-junction blockers CBX, FFA; P2 antagonists SUR and MRS2179; Na^+ ^channel blocker TTX; Ca^2+ ^store depletor CPA with/without added external Ca^2+^. Right - 2-Me-S-ADP evoked Ca^2+ ^transients: Effect of MRS2179. C: Correlation of data obtained through cell number- and fluorescence intensity-based evaluation of Ca^2+ ^transients. The effects of tested compounds/conditions were compared to the control using Mann-Whitney with Bonferroni *post hoc *test (*p < 0.05 and **p < 0.01).

In order to characterise the molecular determinants of ATP-evoked burst-like propagation of Ca^2+ ^transients, effects of different gap-junction inhibitors (CBX, FFA), purinergic P2 antagonists (SUR, MRS2179), agonist 2-Me-S-ADP and Ca^2+ ^store depleting conditions (zero-added Ca^2+ ^with or without CPA) were investigated. To start with, the number of cells participating in the first (100 μM ATP) and the second (100 μM ATP plus test compound) ATP applications were compared (Figure 4A and the Methods section). The changes in the number of ATP responsive cells in the presence of the test compounds (ΔN) were compared to the ΔN values obtained for two consecutive ATP applications both in the absence of test compounds (control), and were given as percentage of the first ATP application (for details of the applied drug testing protocol see also the Methods section). Matching with affinities to those reported for the blockade of intercellular communication through gap junctions [22], ATP-evoked Ca^2+ ^signalling was significantly inhibited by 100 μM and 1 mM CBX (*N *= 14 from 11 rats). FFA applied in 1 mM concentration was also found effective (*N *= 3 from 1 rat, Figure 4A left). The voltage-gated Na^+ ^channel-blocking agent TTX (10 μM, *N *= 4 from 2 rats) and the broad spectrum P2X and P2Y antagonist SUR (100 μM, *N *= 5 from 4 rats; 1 mM, *N *= 3 from 1 rat) were ineffective. However, the ATP puff-evoked Ca^2+ ^transients were virtually eliminated by the selective P2Y_1 _receptor antagonist MRS2179 (100 μM, *N *= 9 from 4 rats) (Figure 4A left). The observed variability in MRS2179 effects most probably reflects the receptor/cell heterogeneity in the NAc slices prepared from 10-14 day old rats. Noteworthy, ATP only activated MRS2179-sensitive P2Y_1 _receptor in the olfactory bulb [23], as well. Locally applied P2Y_1 _selective agonist 2-Me-S-ADP (10 μM, long puff; *N *= 6 from 3 rats) also evoked Ca^2+ ^transients in cells loaded with Fluo-4 acetoxymethyl ester (Fluo-4 AM), indicating the involvement of P2Y_1 _receptor in Ca^2+ ^signalling mechanisms within NAc astrocytes (Figure 4A right). In addition, 100 μM MRS2179 also blocked Ca^2+ ^transients evoked by 2-Me-S-ADP applied as ATP (Figure 4A right, *N *= 6 from 3 rats). Blocking effects of zero-added Ca^2+ ^with (*N *= 8 from 4 rats) or without (*N *= 5 from 4 rats) the intracellular calcium store depleting CPA (10 μM, [18]) or CPA alone (*N *= 5 from 3 rats) (Figure 4A left) suggest that the appearance of concerted Ca^2+ ^transients stimulated by ATP in the NAc slice was reliant on the cellular Ca^2+ ^stores. Ca^2+ ^release from internal stores may also substantiate oscillatory dynamics observed (*see *Figure 2C).

In addition to the number of ATP-responsive cells, we also analysed the magnitude of the response (Figure 4B and Methods section). Maximal dF/F_0 _values for individual cells in the absence and presence of the test compounds were determined. The calculated average change in (dF/F_0_)_max _values were compared to the Δ(dF/F_0_)_max _values obtained for two consecutive ATP applications both in the absence of test compounds (control), and were given as percentage of the first ATP application. This analysis concluded to results consistent with the cell number-based evaluation (Figure 4C). The obtained linear correlation between the cell number- and fluorescence intensity-based data (*R *= 0.94 ± 0.09, Figure 4C) indicates that the Ca^2+ ^transients are produced by an on/off trigger, corroborating Ca^2+ ^store-operated mechanisms.

### SUC activates Ca^2+ ^transients in NAc astrocytes

We tested the hypothesis whether, in addition to ATP, SUC also activates astroglial Ca^2+ ^signals. This was explored in measurements of the effect of SUC alone and in combination with the ATP puff-evoked Ca^2+ ^transients characterized before. To this end, we applied the drug testing protocol described before (*cf*. previous and Methods sections) enabling the detection of the SUC-responsive Ca^2+ ^transients before the second ATP application. This way, the observation of the SUC-responsive cells throughout the second ATP made possible comparison of the spatiotemporal characteristics of the SUC- and ATP puff-evoked Ca^2+ ^transients.

**Figure 5:**
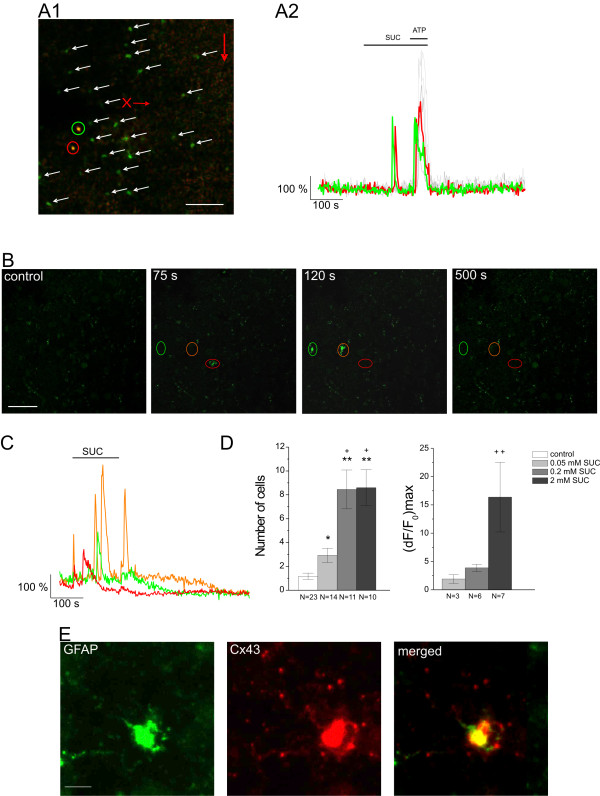
**SUC and ATP evoke overlaying Ca^2+ ^transients in NAc astrocytes**. A1: Confocal image of SUC-responsive cells (green and red circled yellow spots) participated in ATP-stimulated concerted Ca^2+ ^burst (green spots marked by white arrows). Red arrows show directions of SUC (vertical) and ATP (horizontal with x) applications. Scale bar is 50 μm. A2: Representative (dF/F_0_)_max _plots showing effects of 50 μM SUC alone and with 100 μM ATP puff in A1. Red and green traces correspond to the SUC-responsive cells circled in A1 while gray traces show the fluorescence changes of cells marked by white arrows in A2. B: Confocal images showing fluorescence of 5 μM Fluo-4 AM loaded cells in NAc slices during control (left image), 50 μM SUC application (middle images) and washout (right image). Time refers to the fluorescence-time plot (**C**). Scale bar is 50 μm. C: (dF/F_0_)_max _plots showing Ca^2+ ^transients in response to 50 μM SUC in the NAc slice. Each coloured trace corresponds to a Ca^2+ ^transient circled in (B). D: Comparison of cell number- and fluorescence intensity-based summary plots of the effects of SUC on Ca^2+ ^fluorescence in rat NAc slices. *p < 0.05 and **p < 0.01 compared to control, +*p *< 0.05 and ++p < 0.01 compared to 50 μM (Mann-Whitney with Bonferroni *post hoc *tests; [3]). E: SUC-responsive repetitive Ca^2+ ^transients co-localized with astroglial markers. The NAc slice showing SUC-responsive repetitive Ca^2+ ^transients was subsequently double-immunostained for astroglial marker proteins GFAP (green, left) and Cx43 (red, middle) while the observation chamber kept continuously on the stage of the confocal microscope. Our *post-Calcium *protocol (*cf*. Methods section) showed yellow co-localisation of Cx43 and GFAP proteins (right) with a SUC-responsive cell shown in A. Scale bar is 5 μm.

Indeed, superfusion of SUC initiated Ca^2+ ^transients in a sub-population of NAc astrocytes showing Ca^2+ ^transients in response to puffs of ATP (Figure 5A1 left). Apparently, these superimpositions of cells responding to ATP and SUC were independent of the site of activation (*cf*. different localization and direction of ATP puff). The temporal relationships of SUC- and ATP-evoked Ca^2+ ^transients revealed similar dynamics, as shown by representative dF/F_0 _traces (*cf*. Figure 5A2 and Figure 2C). Within the same SUC-responsive cells, Ca^2+ ^transients appeared longer during ATP application (Figure 5A2 red and green traces) conjecturing coincident ATP activation. SUC evoked single (Figure 5A2) and repetitive Ca^2+ ^transients, being more frequent with higher [SUC] (data not shown). Mostly double - but see orange trace in Figure 5C from Figure 5B images at 75 s and 120 s - Ca^2+ ^transients evoked by 50 μM SUC were observed in only about 10% of slices. Similarity of Ca^2+ ^dynamics may suggest that SUC-responsive cells could participate in burst-like Ca^2+ ^signalling evoked by ATP. It can be relevant in this respect that the number of SUC-responsive cells did significantly tend to be higher after the ATP application (1.3 ± 0.3 *vs*. 3.2 ± 0.5 cells, *p *= 0.042, Mann-Whitney U test).

The change in the number of cells showing SUC-evoked Ca^2+ ^transients could be characterised by an EC_50 _value of 50-60 μM (Figure 5D, N = 35 from 16 rats). The same measurements on Ca^2+ ^transients, however, provided less Δ(dF/F_0_)_max _data, the possible reason why the fluorescence intensity-based evaluation of the effective concentration for SUC was less conclusive (Figure 5D). These findings were at variance with the results of the inhibition of ATP puff-evoked Ca^2+ ^transients (*cf*. Figure 4), suggesting that in addition to the oscillatory Ca^2+ ^dynamics, SUC may also alter fluorescence intensity through some other mechanisms making these data more vulnerable to the threshold criterion applied (*cf*. Methods section).

Next, we asked if Ca^2+ ^transients evoked by SUC application were occurred in astrocytes. It has already been demonstrated that the protocol used here for Fluo-4 AM loading preferentially labels astrocytes [3,24]. For further identification of cells displaying SUC-responsive Ca^2+ ^transients we applied double immunostaining for the glial marker proteins GFAP (green, Figure 5E left) and Cx43 (red, Figure 5E middle). Colocalization of Cx43 and GFAP proteins (yellow, Figure 5E right) identified the SUC-responsive cells as astrocytes.

### Neurons within the domain of burst-like Ca^2+ ^signalling are not activated

**Figure 6:**
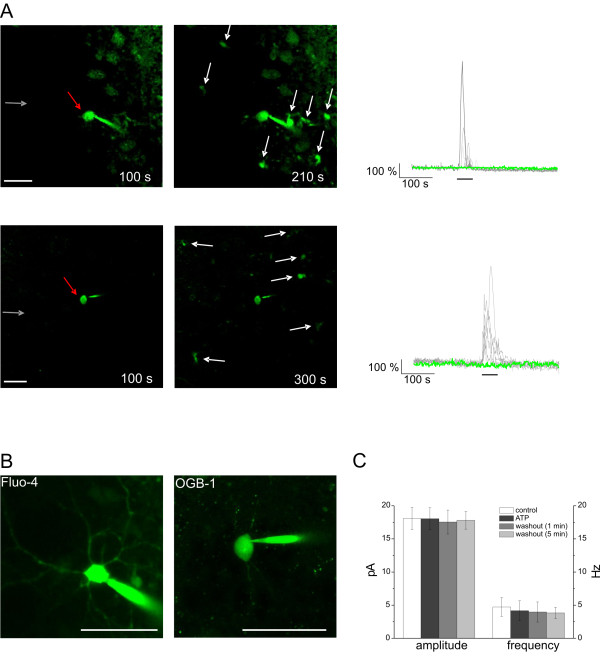
**Neuronal activity is not modified by ATP-responsive astroglial Ca^2+ ^signalling in the NAc slice**. A: Fluo-4/tetrapotassium (200 μM) (upper image, left; red arrow) or OGB-1/hexapotassium (50 μM) (lower image left; red arrow) salt filled neurons (B left or right images) were distinguished by the presence of the fast Na^+ ^current in the NAc slice. They did not colocalize (A upper and lower images, right; gray arrows indicate the orientation of the long ATP puff) with the long ATP puff-induced Ca^2+ ^transients in NAc astrocytes (white arrows). Representative gray and green (dF/F_0_)_max _traces in A show astroglial and neuronal (B) Ca^2+ ^fluorescence, respectively. Scale bar is 50 μm. B: Higher magnification of Fluo-4/tetrapotassium (200 μM) (B left) or OGB-1/hexapotassium (50 μM) (right) filled neurons. Scale bar is 50 μm. C: Summary of the average values of characteristic properties of postsynaptic currents recorded from neurons in the NAc slice. Currents were recorded at -70 mV holding potential using K-Gluconate-based pipette solution. Peak amplitudes and frequencies of individual currents were averaged throughout 1-min intervals. Mann-Whitney test used for statistical evaluation of amplitude and frequency variations, neither of them was found to be significant (18 neurons, 10 slices, 10 rats; *p *> 0.05).

In order to see if *neurons *in the NAc participate in ATP-stimulated astroglial Ca^2+ ^transients, we filled visually identified cells in either the shell or core regions with Ca^2+ ^indicator dyes (200 μM Fluo-4 tetrapotassium salt, [3] or 50 μM Oregon Green 488 BAPTA-1 (OGB-1) hexapotassium salt, [25]). We monitored intracellular Ca^2+ ^changes (Figure 6A green traces) and postsynaptic currents simultaneously. The cells were identified as neurons by the appearance of voltage activated fast Na^+ ^currents during application of a voltage-ramp protocol (-40 mV to +50 mV). This experimental setup allowed us to simultaneously image the Ca^2+ ^signal and monitor the effects of ATP and/or astrocyte Ca^2+ ^signalling on both the post-synaptic currents (*cf*. also Methods section) and the cytosolic Ca^2+ ^of the reporter neuron.

Although the identified reporter neurons were flooded by the long ATP puff (*cf*. Figure 2B right) and surrounded by ATP-responsive NAc astrocytes (Figure 6B left images), astroglial burst-like Ca^2+ ^signalling caused no significant changes in cytosolic Ca^2+ ^(Figure 6A right images; *N *= 15, 11 rats) of these neurons. We measured neuronal Ca^2+ ^(Figure 6A right images and green traces) and spontaneous post-synaptic currents (*i.e*., amplitude and frequency) under control, ATP and distinct washout periods (Figure 6C) and found no significant changes in any of these parameters (Figure 6C; *N *= 18 for 10 rats).

Similarly to ATP application (Figure 6C), postsynaptic currents of neurons within the astroglial Ca^2+ ^signalling network did not exhibit significant changes in control frequency and amplitude (16.3 ± 1.5 pA; 3.0 ± 0.9 Hz) under 50 μM SUC (17.6 ± 2.9 pA, *p *> 0.05; 2.7 ± 1.0 Hz, *p *> 0.05), 50 μM SUC+ATP (18.1 ± 1.8 pA, *p *> 0.05; 2.6 ± 0.8 Hz, *p *> 0.05) and washout (16.4 ± 2.8 pA, *p *> 0.05; 3.0 ± 1.6 Hz, *p *> 0.05) periods (*N *= 3 from 3 rats, data not shown). These findings supported our conclusion that NAc neurons did not participate in astroglial Ca^2+ ^signalling elicited by ATP and/or SUC.

## Discussion

We described that ATP evokes burst-like propagation of Ca^2+ ^signals amongst astrocytes in the NAc. We disclosed for the first time that SUC does also induce Ca^2+ ^transients. We will discuss the mechanisms of Ca^2+ ^signalling amongst astrocytes in the NAc and their possible relation to neuronal activity. The potential importance of metabolites related to brain energy state regulating Ca^2+ ^signals will also be speculated.

### Intercellular Ca^2+ ^signalling through Cx43 containing astrocytes in the NAc

The ATP-evoked burst-like Ca^2+ ^signal apparently propagates through astrocytes coupled by gap-junctions [26-28]. More extensive Ca^2+ ^signalling in response to the localized ATP stimuli in the NAc as compared to nearby brain areas may possibly be due to the more abundant presence of Cx43 protein and gap-junctions thereupon. Our observations also suggest that Cx43 containing gap-junctions participate in burst-like Ca^2+ ^signalling and were expressed only in astrocytes [29]. Note, however, that co-localization of Cx43 and GFAP may also be associated with reactive astrocytes [30] or radial glia [31].

Astroglial burst-like Ca^2+ ^signals are known to propagate *via *two mechanisms [26,27]: transfer of cytosolic inositol (1,4,5)-trisphosphate (IP_3_) directly from cell to cell through gap junction channels and release of ATP onto extracellular purinergic receptors. The CBX-sensitive Ca^2+ ^signal is coupled with the ATP-triggered enhancement of IP_3 _and its diffusion through gap-junction channels between neighbouring astrocytes [32]. Another possible way of CBX- and FFA-sensitive propagation of the Ca^2+ ^signal involves sequential activation of cation channels by ATP that is released through gap-junction hemichannels into the extracellular space [33,34]. These CBX-sensitive Ca^2+ ^waves could be blocked by SUR, a polysulfonated napthylurea used as a broad spectrum antagonist at purine-activated P2X channels and G-protein-coupled P2Y receptors [35-37]. In our experiments SUR (100 μM, 1 mM) did not block the ATP-activated Ca^2+ ^bursts significantly, although it is known to antagonize P2X receptors in the 1-100 μM range [36]. Nevertheless, our results corroborate previous data suggesting that SUR, the widely used antagonist of the effects of ATP at P2 purinoceptors, also induces calcium inducible calcium release (CICR) and increases open channel probability of ryanodine receptor channels [38-42]. These findings can explain the apparent ineffectiveness of SUR as a P2 antagonist by the opposing SUR-induced Ca^2+ ^enhancements through the direct activation of intracellular Ca^2+ ^stores.

In contrast, the blockade of both ATP- and the P2Y_1_-selective 2-Me-S-ADP-evoked Ca^2+ ^transients by the P2Y_1 _selective antagonist MRS2179 indicated that the astroglial burst-like Ca^2+ ^signal may possibly be evoked by activation of the P2Y_1 _receptor subtype in the NAc. The observed variability in MRS2179 effects may be related to developmental changes of P2Y_1 _receptors [43] resulting through receptor/cell heterogeneity within the shell/core regions of NAc slices prepared from 10-14 day old rats. In addition, endogenous SUC may have a co-stimulatory role in ATP-induced astroglial Ca^2+ ^signalling and, by (partially) antagonizing the effects of the P2Y_1 _inhibitor MRS2179 as observed with human platelets [44], may contribute to the inter-slice/rat variability observed. It may be significant in this regard, that co-application of SUC and ATP apparently results in more durable Ca^2+ ^transients, conjecturing coincidence detection.

As discussed before, Ca^2+ ^store mobilization, underlying propagating astroglial burst-like Ca^2+ ^transients can occur via P2Y_1 _receptor activation in the NAc. Both the inhibitor profile (blockade by CBX, FFA and Cx43 antibody) and dynamics of ATP-evoked Ca^2+ ^waves suggest cell-to-cell signalling through the gap-junction-coupled astrocytes in the NAc. Low FFA efficacy may be due to the possible enhancement of intracellular Ca^2+ ^by FFA acting at type 6 transient receptor potential canonical channel (TRPC6) [45,46].

As outlined above, the alternative ATP-driven way of signal propagation [26,27,32-34] may not be excluded purely on the basis of the apparent lack of SUR blockade of ATP-evoked Ca^2+ ^signals. Binding of CBX to SUC receptor [4-6], however, could be accounted for some blockade of ATP-driven Ca^2+ ^signal propagation as detailed below.

### NAc astrocytes respond to SUC

SUC binding sites have previously been disclosed in rat forebrain and human NAc membrane homogenates [4,6], however, their functions have not been assigned yet. Identity of SUC-sensitive GHB [5] and GHB-sensitive SUC [4] binding sites raised the possibility to relate SUC and different GHB actions [47-50]. Importantly, the binding site of GHB/SUC interacts with CBX [4-6], a blocker of gap-junctions that are major players of astroglial Ca^2+ ^signalling [51]. We have also shown previously that GHB activates intracellular store-dependent Ca^2+ ^transients in NAc astrocytes [3]. Here we report, that SUC induced repetitive Ca^2+ ^transients occur in a subpopulation of Cx43^+ ^and GFAP^+ ^cells responding to ATP with burst-like Ca^2+ ^signals involving gap-junctions. SUC may bind to a purinergic G protein coupled receptor (GPCR) subtype or some other SUC-responsive membrane receptor such as SUCNR1 (GPCR91). Interestingly, the SUCNR1 gene is located on chromosome 3 as part of a cluster of seven GPCRs in close vicinity to the genes for P2Y_1 _[52]. Also, direct effect of SUC on gap-junctions cannot be excluded. The SUCNR1 has recently been shown to regulate cellular functions implicated in renal blood pressure regulation [44,53] and lipolysis of white adipose tissue [54]. In addition, the SUCNR1 may also have a role for immunity, hyperglycemia, retinal neovascularization, ischemic liver injury and hematopoiesis as reviewed recently [44]. The presence of SUCNR1 has been demonstrated in human platelets and megakaryocytes [44], in various cells of distal nephron [55] and in cardiomyocytes [56].

The concentration of SUC in plasma [57-59] increases with exercise [60], metabolic acidosis [61], hypertension and metabolic diseases [62] from 5 μM up to 125 μM. These data suggest that the tissue concentration of SUC can be high enough to induce astroglial Ca^2+ ^transients characterised by the EC_50 _value for SUC-responsive cells within the range of 50-60 μM. Using arachidonic acid, ADP and SUC as platelet agonists, aggregation in response to SUC alone was highly variable with only 29% of donors showing a (mostly delayed) platelet response [44]. In contrast, SUC reproducibly and concentration-dependently enhanced platelet aggregation in response to low concentrations of exogenous ADP [44]. Assuming the presence of SUCNR1 and P2Y_1 _in astrocyte membrane, we conjecture co-stimulatory roles played by endogenous SUC and ATP within the brain. Such a coincidence detection performed by astrocytes could explain why astrocytic Ca^2+ ^transients may be dramatically affected by pathological conditions associated with intense neuronal firing [63].

### Signalling layer independent of neurones

It has been reported, that mechanically evoked astrocytic Ca^2+ ^waves mediated by the release of ATP and the activation of P2 receptors in hippocampal cultures down-regulate excitatory glutamatergic synaptic transmission in an ATP-dependent manner [64]. Using astrocyte-specific inducible transgenic mice (dnSNARE mice), it has been evidenced that by the release of ATP, which accumulates as adenosine, astrocytes tonically suppress synaptic transmission in acutely isolated hippocampal slices [65]. Causal linkage between reduced activation of adenosine receptors and surface expression of NMDA receptors has also been evidenced [[66] and references cited there]. We assumed therefore, that by using ATP puff, which may also reduce activity of NAc neurons, we could isolate 'autonomous' astrocytic Ca^2+ ^signalling. Indeed, the loading pattern of the Ca^2+ ^indicator Fluo-4 AM [3,24] and the presence of Cx43 protein in cells responding to SUC/ATP by Ca^2+ ^transients identified them as astrocytes.

Propagation of the Ca^2+ ^signal in ellipsoidal and/or radial waveform at a speed of approximately 10 μm/s in NAc astrocytes is comparable to the time evolution of Bergmann glia Ca^2+ ^transients, as previously recorded from fixed location in vitro [9,10] and in vivo [7,13]. The similarity suggests both phenomena involve ATP-triggered release of Ca^2+ ^from intracellular stores, as found. The type of glial Ca^2+ ^signalling classified as bursts persisted after application of TTX in the cerebellar Bergmann glia in vivo [7] and NAc astrocytes in vitro (this work). Unchanged postsynaptic currents and an invariable cytosolic Ca^2+ ^level of neurons within the astrocyte network that exhibited concerted Ca^2+ ^bursts support our conclusion that the ATP evokes astroglial burst-like Ca^2+ ^signals independently of neuronal activity in the NAc. In this respect, ATP/SUC-evoked astrocytic Ca^2+ ^transients differ from the ones dependent on the metabotropic Glu recptor subtype 5, leading to the activation of the NR2B subunit containing N-Me-D-Asp receptors (NMDARs) of medium spiny neurones in the NAc or the striatum [17]. Our conclusion upholds previous findings by us [3] and others [[28], reviewed in [63]], suggesting the existence of "autonomous" activation of astroglial Ca^2+ ^signals. Remarkably, glial Ca^2+ ^bursting activity observed in the cerebellum of awake, behaving animal is also found to be independent of neuronal activity [7].

Findings obtained with the NAc model of glia activation described in this work do not exclude, however, that Ca^2+ ^signalling involving ATP/SUC sensing cells might be a unique feature of the NAc. We may also assume such a signalling route in other brain areas exhibiting extensive Cx43 expression. The way of Ca^2+ ^signalling is probably characteristic of a given brain region [28]. Ca^2+ ^bursts have been shown in cultured astrocytes [34], white matter tract [67], cerebellum [7] and cortical brain slices [28,68,69]. Ca^2+ ^bursts in the neocortex depend on gap-junction coupling of astrocytes and are not influenced by neuronal activity while in the corpus callosum Ca^2+ ^signalling requires ATP-release mechanism but not gap-junction expression [28]. These pathways are not independent from each other and most probably linked to the cell types participated in Ca^2+ ^signalling [19]. In the retina, astrocytic Ca^2+ ^wave is dependent on gap-junctions, while the wave propagation between astrocytes and Müller cells requires ATP-release mechanism [70]. The expression of the major gap-junction protein of astrocytes Cx43 influences purinergic receptor expression in the spinal cord astrocytes [71]. These examples rather support the view that glial Ca^2+ ^bursts may represent a signalling layer independent of neurons.

The issue of independence as opposed to interdependence of glial and neuronal activities is related to the question of astrocytic release of signalling molecules that may also modulate synaptic transmission, a process called gliotransmission. As outlined before, there are several indications, that astrocytes do affect synaptic activity through the release of ATP, Glu, D-Ser, tumour necrosis factor alpha [[63-66,72] and references cited] or uptake of Glu [73,74] and GABA [74]. It is to note, that the physiological relevance of gliotransmission in long-term potentiation (LTP) was called into question [[75,76], *see *also [77] for additional references]. In contrast, Ca^2+^-dependent release of D-Ser from an astrocyte affecting NMDAR-dependent plasticity was demonstrated by clamping internal Ca^2+ ^in individual astrocytes of the CA1 area of the hippocampus [77].

## Conclusions

Diverse cellular pathways of astroglial Ca^2+ ^signals are differently pronounced in various brain regions and they can also co-occur. Besides, the initial stimuli are likely a determinant of the relevant physiological function being involved.

The findings of the present study describe for the first time the Ca^2+ ^signals evoked in astrocytes of the brain reward area, the nucleus accumbens, by the citric acid cycle intermediate SUC. We also identify these SUC-responsive Ca^2+ ^signals in ATP-evoked burst-like intercellular Ca^2+ ^signalling. We provide evidence that ATP- and SUC-responsive glial Ca^2+ ^signals are independent on neuronal activity therefore apparently represent a signalling layer independent of NAc neurons.

ATP-induced calcium responses have previously been shown in cortical and hippocampal astrocytes whereas succinate-induced calcium signals had not been described in astrocytes. This previously unrecognized glial action of the major cellular energy metabolite SUC may represent a link between brain energy states and Ca^2+ ^signalling in astrocytic networks.

## Methods

### Ethics statement

Animal care and preparations were in accordance with the Helsinki declaration, the European Council Directive of 24 November 1986 (86/609/EEC), the Hungarian Animal Act 1998, and were associated local guidelines, as approved by the Institutional Animal Care and Use Committee (approval ID: MÁB 1.51.4.).

### Buffers and test compounds

Slice preparing buffer contained in mM: 250 sucrose, 2 KCl, 1.25 KH_2_PO_4_, 10 MgSO_4_, 2 CaCl_2_, 16 NaH_2_CO_3 _and 10 glucose. Artificial cerebrospinal fluid (ACSF) contained in mM: 129 NaCl, 2 KCl, 1.25 KH_2_PO_4_, 1 MgSO_4_, 2 CaCl_2_, 16 NaHCO_3 _and 10 glucose.

The following drugs were applied *via *the ACSF perfusion: SUC, GHB, CBX, another gap-junction blocker FFA (purchased from Sigma-Aldrich, Budapest, Hungary); P2X/P2Y receptor antagonist SUR, the P2Y_1 _receptor antagonist MRS2179, the endoplasmic Ca^2+^-ATPase inhibitor CPA, and TTX (from Tocris, Bristol, UK). Agonists ATP and 2-Me-S-ADP were locally applied through a glass micropipette (Sigma-Aldrich, Budapest, Hungary).

Fluorescence indicators Fluo-4 AM, Fluo-4 tetrapotassium salt, OGB-1 hexapotassium salt, propidium iodide (PI), SR101 and pluronic acid were purchased from Molecular Probes (Eugene, USA). Stock solutions of ester fluorescence indicators prepared in DMSO were diluted to 0.1% DMSO in the staining solution.

### Acute slice preparation and dye loading

Coronal slices from the forebrain through the NAc and the caudate putamen (CP) were prepared for the imaging experiments. Young, 10-14 day-old male Wistar rats were decapitated and the brains were quickly removed. The forebrains were serially cut into 300 μm thick coronal sections (Vibratome, Technical Products International Inc., St. Louis, MO, USA). The slices were collected in ice-cold preparation buffer and incubated for one hour under humidified gas-mixture carbogen (5% CO_2 _+ 95% O_2_) atmosphere in an interface-type holding chamber containing warmed (35°C) ACSF. After preincubation in 2% pluronic acid containing ACSF for 2 minutes, slices were further incubated with 5 μM Fluo-4 AM in ACSF at 35°C in the dark under humidified carbogen (5% CO_2 _+ 95% O_2_) atmosphere, for one hour [3]. In order to monitor cell death, several slices were exposed to double dye-loading protocol, performed by adding 7.5 μM PI (excitation: 534 nm, emission: 570-600 nm) to the Fluo-4 AM containing ACSF. In order to allow for cleavage of the AM ester group of Fluo-4, slices were transferred to dye-free ACSF at least 30 minutes before the start of the experiment [78].

### Confocal imaging and drug testing protocol

We used Fluo-4 AM for quantifying astroglial Ca^2+ ^concentrations in the 100 nM to 1 microM range [3]. Fluo-4 offers high fluorescence emission due to its greater absorption near 488 nm, and a large dynamic range for reporting [Ca^2+^] around a K_d_(Ca^2+^) of 345 nM [79]. Fluorescence recordings of changes in the intracellular Ca^2+ ^ion level in cells loaded with Fluo-4 AM were performed, with an upright epifluorescent microscope (Olympus BX61WI, Olympus, Budapest, Hungary) equipped with the FluoView300 confocal laser-scanning system (Olympus, Budapest, Hungary) using 20× (0.5 numerical aperture) or 60 (0.9 numerical aperture) water immersion objectives (for details, *see *[3]). Image acquisition rate was controlled by a computer running Tiempo software for FluoView300 (Olympus, Budapest, Hungary).

Freshly isolated slices were transferred to the submerge-type recording chamber mounted on the stage of the microscope and were superfused with carbogenated (5% CO_2 _+ 95% O_2_) ACSF (3 ml/min, room temperature). Serial scanning of slices were made at 488 nm excitation wavelengths and emitted green fluorescence was collected through a 510-530 nm bandpass filter. Ca^2+ ^transients were initiated by application of SUC in the perfusion or by pressure-ejection of 100 μM ATP in ACSF through a glass micropipette (5-10 μm diameter) on the slice surface. Fluorescence intensity changes within a 355 × 355 μm field containing approximately 100 Fluo-4 AM loaded cells around the area of the ATP puff, were followed over a 10-minute interval (2 s/image).

**Figure 7:**
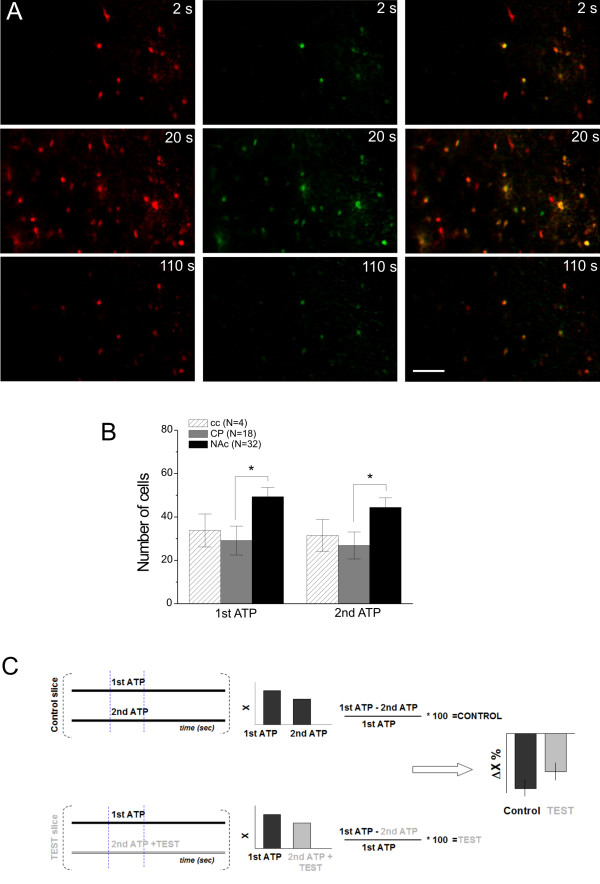
**Ca^2+ ^transients evoked by consecutive ATP puffs applied onto the surface of the NAc slice**. A: Time series of Ca^2+ ^transients in response of the 1st (left, red) and the 2nd (middle, green) ATP-puff applied by pressure-ejection of 100 μM ATP through a 5-10 μM diameter patch-pipette for 60 s (long puff). Time refers to the time elapsed from the beginning of the ATP applications. Overlaying Ca^2+ ^transients (right, yellow) suggest similarity of Ca^2+ ^dynamics of consecutive ATP puffs. Scale bar is 50 μm. B: Comparison of Ca^2+ ^signalling after the 1st and 2nd ATP application in the corpus callosum (*N *= 4), caudate putamen (*N *= 18) and NAc (*N *= 32). Data represent means ± S.E.M. (Mann-Whitney with Bonferroni *post hoc *test: *p < 0.05. C: The protocol applied for testing effects of different drugs/conditions on ATP-stimulated Ca^2+ ^elevations in slices from the NAc. The difference between the two recordings in the number of cells (ΔN) or maximal fluorescence intensity Δ(dF/F_0_)_max _was expressed as the percentage of the effects of the first (control) stimulus (100 μM ATP or 10 μM 2-Me-S-ADP) application.

Two 10-minute-long consecutive recordings were made from each slice with a 20-minute resting time between the two recordings. ATP was applied 3 min after the beginning of each 10-minute-long recording period. All tested drugs (CBX, FFA, SUR, MRS2179, CPA, TTX) and/or zero-added Ca^2+ ^condition were applied throughout the second 10-minute-long recording period and were present at the time of the second ATP application. Co-localisation of Ca^2+ ^transients evoked by the 1st and the 2nd ATP puffs indicated similarity of Ca^2+ ^dynamics (Figure 7A), making them strictly comparable. The number of cells showing Ca^2+ ^increase at approximately 15-30 μm below the surface of the NAc slice was also similar in consecutive ATP applications (44 ± 25 cells, Figure 7B).

Images taken during the ATP application were summed and the average of images taken during the control period was subtracted as a background. The Fluo-4 loaded cells were identified by using custom written algorithms in Matlab under strict visual control. The intensity changes in individual cells were calculated as dF/F_0_. To identify ATP responsive cells, the standard deviances of the control periods of the dF/F_0 _traces (SD_control_) were calculated and the traces with (dF/F_0_)_max,ATP _> 5*SD_control _were selected. The number of cells with fluorescence intensity changes above the threshold was counted to determine the number of responsive cells (N) and the average of their maximal dF/F_0 _values ((dF/F_0_)_max_) during the ATP application period was used to assess the magnitude of the response. The changes in N and (dF/F_0_)_max _values (ΔN and Δ(dF/F_0_)_max_, respectively) obtained in the presence of test compounds were normalized to the corresponding N and (dF/F_0_)_max _values obtained in the absence of the test compounds (Figure 7C). We observed that the number of ATP responsive cells and their maximal response were reduced in the second ATP application even if the test compounds were not present. Therefore to take this reduction into account, the calculated ΔN and Δ(dF/F_0_)_max _values for each condition were compared (ANOVA) to ΔN and Δ(dF/F_0_)_max _values obtained for two consecutive ATP applications both in the absence of test compounds (control in Figure 4).

Data presented are mean ± S.E.M., with N denoting the number of slices in a given experimental condition. Statistical analysis was performed using the non-parametric Mann-Whitney test with Bonferroni *post hoc *test (OriginLab Co., Northampton, UK) and p < 0.05 was considered statistically significant. If otherwise stated, the effects of different treatments were compared to the control.

### Electrophysiology

Whole cell patch clamp recordings were performed both in the shell and the core areas of the NAc by using a MultiClamp 700A amplifier (Axon CNS, Molecular Devices, Sunnyvale, California, USA). Signals were low-pass filtered at 2 kHz and digitized at 10 kHz (Digidata1320A, Axon Instruments). Cells selected by their visual appearance were identified as neurons by the presence of voltage activated fast Na^+ ^currents during application of a voltage ramp protocol -40 to +50 mV (pClamp8, Axon Instruments). 6-8 Mc pipettes pulled from borosilicate glass capillaries were filled with an intracellular solution (containing in mM: 135 K-Gluconate, 10 NaCl, 0.05 CaCl_2_, 2 adenosine-triphosphate Mg^2+ ^salt, 10 4-(2-hydroxyethyl)piperazine-1-ethanesulfonic acid (HEPES); pH 7.3 (set with 1N KOH). In order to visualize and monitor cytosolic Ca^2+ ^of patched neurons, 200 μM Fluo-4 tetrapotassium salt or 50 μM OGB-1 hexapotassium salt (excitation: 488 nm, emission: 510-530 nm for both dyes) was added to the intracellular solution. Cells (input resistance: 166 ± 13 MΩ) were clamped to -70 mV without corrections for liquid junction potential (-15 mV). At this potential inward spontaneous postsynaptic currents were recorded. If signs of seal deterioration or cell closure occurred, the recordings were discarded. The first 10 min of each recording were used for monitoring stabilization of the baseline. Then, a 1-minute duration control period, followed by ATP application (1 min), and a washout period (5-10 min) were recorded.

Spontaneous postsynaptic currents (PSCs) were analyzed using Mini Analysis software (Synaptosoft, Decatur, USA; http://www.synaptosoft.com) in 4 data segments (1 min, each) recorded prior to, at the ATP application, washout after ATP application and late-washout periods. The automatically detected events (threshold: 10 pA) were verified by visual inspection. Multiple comparisons were done with the non-parametric Mann-Whitney test (OriginLab Co., Northampton, UK). Statistical significance was defined as p < 0.05. The data are presented as mean ± S.E.M.

### Tissue processing for immunohistochemistry

Adult, male Wistar rats (n = 3) (200-250 g body weight; Charles Rivers Laboratories, Hungary) were deeply anesthetized with a mixture containing 0.2 ml/300 g b.w. ketamine (100 mg/ml) and 0.2 ml/300 g b.w. xylazine (20 mg/ml), and perfused transcardially with 150 ml saline followed by 300 ml of ice-cold 4% paraformaldehyde in phosphate buffer, pH 7.0 (PB). Brains were removed and postfixed in the same fixative solution for 24 h, and transferred to PB containing 20% sucrose for 2 days. Serial coronal sections were cut at 50 μm on a sliding microtome (SM 2000R, Leica Microsystems, Nussloch, Germany) between +4.0 mm to 0 mm from the level of the bregma. Sections were collected in PB containing 0.1% sodium azide and stored at 4°C until further processing.

### Cx43 immunohistochemistry

Free-floating sections were immunolabeled for Cx43 using an affinity-purified rabbit polyclonal antiserum raised against the carboxy terminal 362-382 peptide segment KPSSRASSRASSRPRPDDLEI of Cx43 (Abcam, Cambridge, UK, catalogue number: ab11370). Brain sections were pretreated in PB containing 0.5% Triton X-100 and 3% bovine serum albumin for 1 h. Then, they were incubated with a primary antibody against Cx43 (1:1250) in PB containing 0.5% Triton X-100 and 3% bovine serum albumin and 0.1% sodium azide for 48 h at room temperature. Sections were then incubated in biotin-conjugated donkey anti-rabbit secondary antibody at 1:1000 (Jackson Immunoresearch, West Grove, PA) for 2 h, followed by incubation in avidin-biotin-horseradish peroxidase complex (ABC) at 1:500 (Vectastain ABC Elite kit, Vector Laboratories, Burlingame, CA) for 2 h. Then, sections were treated with either 0.02% diaminobenzidine (Sigma) or fluorescein isothiocyanate (FITC)-tyramide (1:8000) with 0.003% H_2_O_2 _in Tris-HCl buffer (0.05 M, pH 8.2) for 6 min. After washing, the sections were mounted on positively charged slides (Superfrost Plus, Fisher Scientific) and coverslipped with either Cytoseal 60 (Stephens Scientific, Riverdale, NJ, USA) or in antifade medium (Prolong Antifade Kit, Molecular Probes).

### Cx43 and GFAP double immunohistochemistry

Free-floating sections were first immunolabeled for Cx43 using FITC-tyramide amplification immunohistochemistry, as described above. Then, sections were incubated overnight in a mouse monoclonal anti-GFAP, a marker of astrocytes (1:250; catalogue number: sc-33673, Santa Cruz Biotechnology, Santa Cruz, CA). Subsequently, sections were incubated in Alexa 594 donkey anti-mouse secondary antibody (1:500; Molecular Probes) for 2 h, then mounted and coverslipped, as described above. Sections were examined using an Olympus BX60 light microscope also equipped with fluorescent epi-illumination. Images were captured at 2048 × 2048 pixel resolution with a SPOT Xplorer digital CCD camera (Diagnostic Instruments, Sterling Heights, MI) using 4-20× objectives. Confocal images were acquired with a Nikon Eclipse E800 confocal microscope equipped with a BioRad Radiance 2100 Laser Scanning System using 20-60× objectives (at an optical thickness of 1-3 μm). Contrast and sharpness of the images were adjusted using the "levels" and "sharpness" commands in Adobe Photoshop CS 8.0. Colours were adjusted so that Cx43 appeared red and GFAP was green. Full resolution was maintained until the photomicrographs were cropped and assembled for printing, at which point images were adjusted to a resolution of 300 dpi.

### Post-calcium imaging immunohistochemistry protocol for Cx43 and GFAP

In order to identify the cell types involved in Ca^2+ ^bursts, the brain slices were immunostained with antibodies for astrocyte marker proteins (Cx43 and GFAP). However, Fluo-4 signal could not be preserved through fixation with either 0.4% paraformaldehyde or 40 mg/ml EDAC (1-ethyl-3-(3-dimethylaminopropyl)-carbodiimide, Sigma-Aldrich; Dawling and Deitmer, 2002). Therefore, co-localization between SUC/ATP-responsive cells and cell type markers could not be resolved. Hence we opted to perform the *in situ *immunostaining in non-fixed slices directly after the Ca^2+ ^imaging protocol. Slices used previously to measure Ca^2+ ^changes in response to SUC/ATP application were treated as follows: upon completion of the calcium-imaging experiments, each slice was kept in its original position in the recording chamber (using a ballast) and incubated with Cx43 (1:300) and GFAP (1:200) primary antibodies for 30 min, at room temperature. After 3 × 10 minute washing in ACSF, the slice was incubated with Chromeo 546 goat anti-rabbit (1:100 Abcam, Cambridge, UK, catalogue number: ab60317) secondary antibody and Alexa 488 donkey anti-mouse (1:100, Molecular Probes) secondary antibody in ACSF for 30 min, at room temperature. This was followed by 3 × 10 minute washing in ACSF. Serial Z-scans of Cx43- (excitation: 543 nm, emission: 570-600 nm) and GFAP- (excitation: 488 nm, emission: 510-530 nm) labelled slices were acquired between the slice surface and the maximal penetration depth of the antibodies (approximately 60-70 μm from the surface) through a 20× objective (1 μm/step). Since the fluorescence emission of both Fluo-4 and Alexa-488 dyes are collected in the 510-530 nm range, GFAP-specific staining was obtained by subtracting the Fluo-4 fluorescence from the GFAP immunolabelling signal, at each Z depth. Optical sections from identical depths of Fluo-4 and Cx43 images were merged along the Z axis. Single cells and glia filaments that showed double immunolabelling were recorded through a 60× objective and Z-scans were performed by alternating the excitation wavelengths between 543 nm (Cx43) and 488 m (GFAP) using Tiempo software for FluoView300, at each depth (0.1 μm/step). Images were processed using ImageJ 1.44 [80] and Adobe Photoshop CS 8.0 image analysis software.

## Authors' contributions

TM carried out the calcium imaging studies and drafted the manuscript. TM and LH performed the statistical analysis. AD carried out the immunoassays. GNY performed the electrophysiological studies. PB participated in the design of the study and data analysis. LH, EZS and MP participated in the interpretation of data and helped to draft the manuscript. JK conceived of the study, and participated in its design and coordination and helped to draft the manuscript. All authors read and approved the final manuscript.

## Supplementary Material

Additional file 1 **Ca^2+ ^signalling evoked by ATP in the acute NAc slice from the rat brain**. Movie of time measurement showing ATP (100 μM) application onto a Fluo-4 AM loaded acute NAc slice (300 μm). ATP was applied for 60 s (long puff) through a glass micropipette right above the tissue surface. Image acquisition frequency was 2 s in depth of 25 μm from the slice surface. Olympus FluoView300 software collected .tiff file stacks were coloured and converted to .avi file by ImageJ 1.44 (32-bit) image processing and analysis software [80]. ATP-responsive cells were counted by using an ImageJ macro developed to average stacks before the time of ATP application and to subtract this average from every stack of the 10 minute-long recording. In this way, we got a 10-minute-long movie showing only the cells that had a fluorescence intensity change. Click here for file
